# Increased thalamic glutamate/glutamine levels in migraineurs

**DOI:** 10.1186/s10194-018-0885-8

**Published:** 2018-07-17

**Authors:** Adina Bathel, Lauren Schweizer, Philipp Stude, Benjamin Glaubitz, Niklas Wulms, Sibel Delice, Tobias Schmidt-Wilcke

**Affiliations:** 1 Department of Neurology, Berufsgenossenschaftliches Universitätsklinikum Bergmannsheil, Ruhr-University-Bochum, Bochum, Germany; 2 Department of Anesthesiology, Unfallkrankenhaus Berlin, Berlin, Germany; 3 Department of Neurology, St. Mauritius Therapieklinik, Meerbusch, Germany; 4 Institute of Clinical Neuroscience and Medical Psychology, Universitätsklinikum Düsseldorf, Düsseldorf, Germany

**Keywords:** Migraine, Headache, Thalamus, Occipital cortex, GABA, Spectroscopy

## Abstract

**Background:**

Increased cortical excitability has been hypothesized to play a critical role in various neurological disorders, such as restless legs syndrome, epilepsy and migraine. Particularly for migraine, local hyperexcitability has been reported. Levels of regional excitatory and inhibitory neurotransmitters are related to cortical excitability and hence may play a role in the origin of the disease. Consequently, a mismatch of the excitatory-inhibitory neurotransmitter network might contribute to local hyperexcitability and the onset of migraine attacks. In this study we sought to assess local levels of glutamate / glutamine (GLX) and gamma-aminobutyric acid (GABA) in the occipital cortex and right thalamus of migraineurs and healthy subjects.

**Methods:**

We measured interictally local biochemical concentrations in the occipital lobe and the right thalamus in patients with migraine (without aura) and healthy controls (HCs) using proton magnetic resonance spectroscopy at 3 T. GLX levels were acquired using PRESS and GABA levels using the GABA-sensitive editing sequence MEGA-PRESS. Regional GLX and GABA levels were compared between groups.

**Results:**

Statistical analyses revealed significantly increased GLX levels in both the primary occipital cortex and thalamus. However, we found no group differences in GABA levels for these two regions. Correlation analyses within the migraine group revealed no significant correlations between pain intensity and levels of GLX or GABA in either of the two brain regions.

**Conclusions:**

Further research is needed to investigate the role of GABA/GLX ratios in greater depth and to measure changes in neurotransmitter levels over time, i.e. during migraine attacks and interictally.

## Background

Migraine is one of the most common neurological disorders, thought to affect up to 6% of men and 18% of women in western industrialized countries [1]. Apart from severe headaches, migraine patients often experience additional symptoms such as nausea and phono- and photophobia, strongly suggesting a multisystem involvement of the disease. The underlying mechanisms of migraine attacks, however, are not fully understood and different pathophysiological mechanisms have been proposed, such as dysfunction of the nucleus coeruleus [2], sterile neurogenic inflammation of the meninges [3] and cortical spreading depression [4]. Genetic studies have exposed a potential involvement of both glutamatergic and GABAergic receptors [5–8]. Additionally, there has been evidence of interictal hyperexcitability, e.g. in the visual cortex [9–12], suggesting a mechanistic role of an excitatory-inhibitory dysbalance contributing to the pathophysiology of migraine. However, the specific roles of glutamate and glutamine (GLX) on the one hand and gamma-amino-butyric acid (GABA) on the other hand, the major excitatory and inhibitory neurotransmitters in the brain, respectively, remain to be fully elucidated.

In vivo measurement of neurometabolite concentrations in the brain remains challenging; standardized clinical techniques, such as lumbar puncture and cerebrospinal fluid (CSF) analysis exclude the possibility to link abnormal metabolite levels to specific regions, but instead generalize the pathophysiology to the entire CNS. In vivo proton magnetic resonance spectroscopy (^1^H-MRS), however, can be used to measure local concentrations of neurometabolites in the human brain non-invasively. Recent studies have investigated altered neurometabolite levels in migraine patients during interictal periods and have presented evidence of both, increased GLX levels in the occipital cortex [12, 13] as well as altered GABA levels in the occipital and posterior cingulate cortices [14, 15]. GABA levels in the occipital cortex were shown to be either reduced in migraine patients as compared to HCs [15] or unchanged, but with an association between GABA levels and disease severity, such that lower levels were related to higher disease severity [16]. Using a more GABA-sensitive editing sequence, Mescher-Garwood-Point Resolved Spectroscopy (MEGA-PRESS), Aguila and colleagues could show that migraine patients also displayed altered GABA in the posterior cingulate cortex, in this case increased levels [14].

Apart from the occipital and cingulate cortices there are several other brain regions that have implications in the pathophysiology of migraine, but also in pain in general. One of these regions is the thalamus, a subcortical brain structure that is tightly coupled in a bidirectional manner with the cortex. The thalamus is best known for its role in relaying almost all sensory modalities to the brain [17, 18]. The presence of multi-sensory symptoms during migraine attacks and the central role of thalamic indicate a potential involvement of the thalamus during the attacks themselves. Interestingly Noseda et al. could recently demonstrate the direct effect of GLX and GABA on the activity of thalamic trigeminovascular neurons [19]. However, to our knowledge, no studies have been performed that specifically looked at thalamic GLX and GABA levels in migraine patients.

Using ^1^H-MRS, and specifically the MEGA-PRESS technique, we investigated migraine patients (without aura) and HCs to determine whether thalamic and occipital GLX and GABA levels are altered in migraine patients, and whether local transmitter concentrations relate to any pain features. Migraine patients were scanned interictally at least three days after their last attack and two days before the next. We hypothesized that migraine patients would show a shift towards an increased excitatory tone, either in terms of increased GLX or decreased GABA levels.

## Methods

### Subjects

Migraine patients were recruited consecutively from the headache-outpatient clinic of the Neurology Department of the University Hospital Bergmannsheil Bochum. Study participants provided informed written consent prior to study enrollment. The local ethics committee had approved of the study (No. 4823–13). Migraine was diagnosed by an experienced neurologist (PS) according to the revised criteria for migraine of the International Headache Society [20]. To participate in the study migraineurs had to report (prior to enrolment) a migraine frequency of at least two migraine attacks per month. Only patients with migraine without aura were included. Participants were therapy naïve, or paused a consisting migraine prophylaxis 14 days prior to the MR scan. Only patients who had been pain free for at least 72 h prior to scanning and 48 h thereafter were enrolled. Migraine patients were compared to HCs. HCs had no personal or family history of migraine. Participants were excluded if they were taking medication affecting CNS metabolism (e.g. anticonvulsant/antidepressant medications) or had a history of neurological or psychiatric diseases, e.g. multiple sclerosis, schizophrenia, or mood disorders. All subjects underwent a thorough neurological examination. Patients additionally rated pain intensity during the last two attacks and completed the Migraine Disability Assessment (MIDAS). Since depression frequently co-occurs with migraine, we also screened for depressive symptoms. Patients filled out the Beck depression inventory questionnaire (BDI). Only patients with BDI scores < 20 (where 20 or more indicate the presence of a moderate or severe depression) were included. For details see Table 1.

**Table 1:** Study cohort

Study#	Gender	Age	Maximum Pain Intensity	Average Pain Intensity	Number of Days with Headache (last 3 months)	MIDAS Grade	BDI Score
1	F	39	7	7	3	3	7
2	F	23	6	6	27	4	1
4	F	29	7	7	40	4	6
6	M	32	10	5	4	3	3
7	M	35	7	7	18	3	3
9	M	45	7	7	3	4	11
10	F	39	4	4	6	3	5
15	F	43	5	5	5	3	0
16	F	31	8	8	5	3	0
19	F	26	8	6	20	3	1
20	F	36	8	8	35	4	2
21	F	32	9	8	12	4	16
48	F	24	8	7	6	2	2
49	F	66	8	8	20	4	4
50	F	29	3	3	2	1	0

Clinical descriptors: *BDI* Beck depression inventory, *F* female, *M* male, *MIDAS* Migraine Disability Assessment

### Spectroscopy

**Fig. 1:**
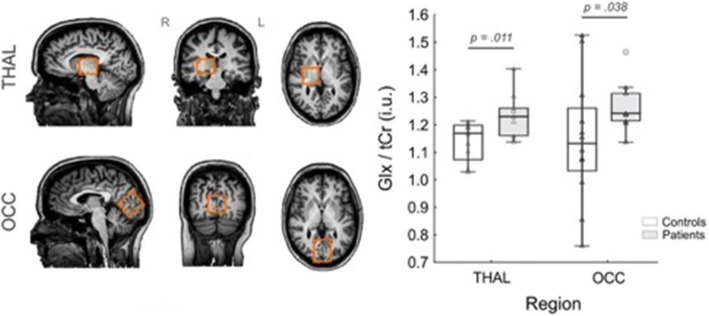
Placement of spectroscopy voxels. Abbreviations: Cr = creatine, GLX = glutamate/glutamine, OCC = occipital cortex Thal = thalamus. Orange box depicts voxel placement in the thalamus and occipital cortex

**Fig. 2:**
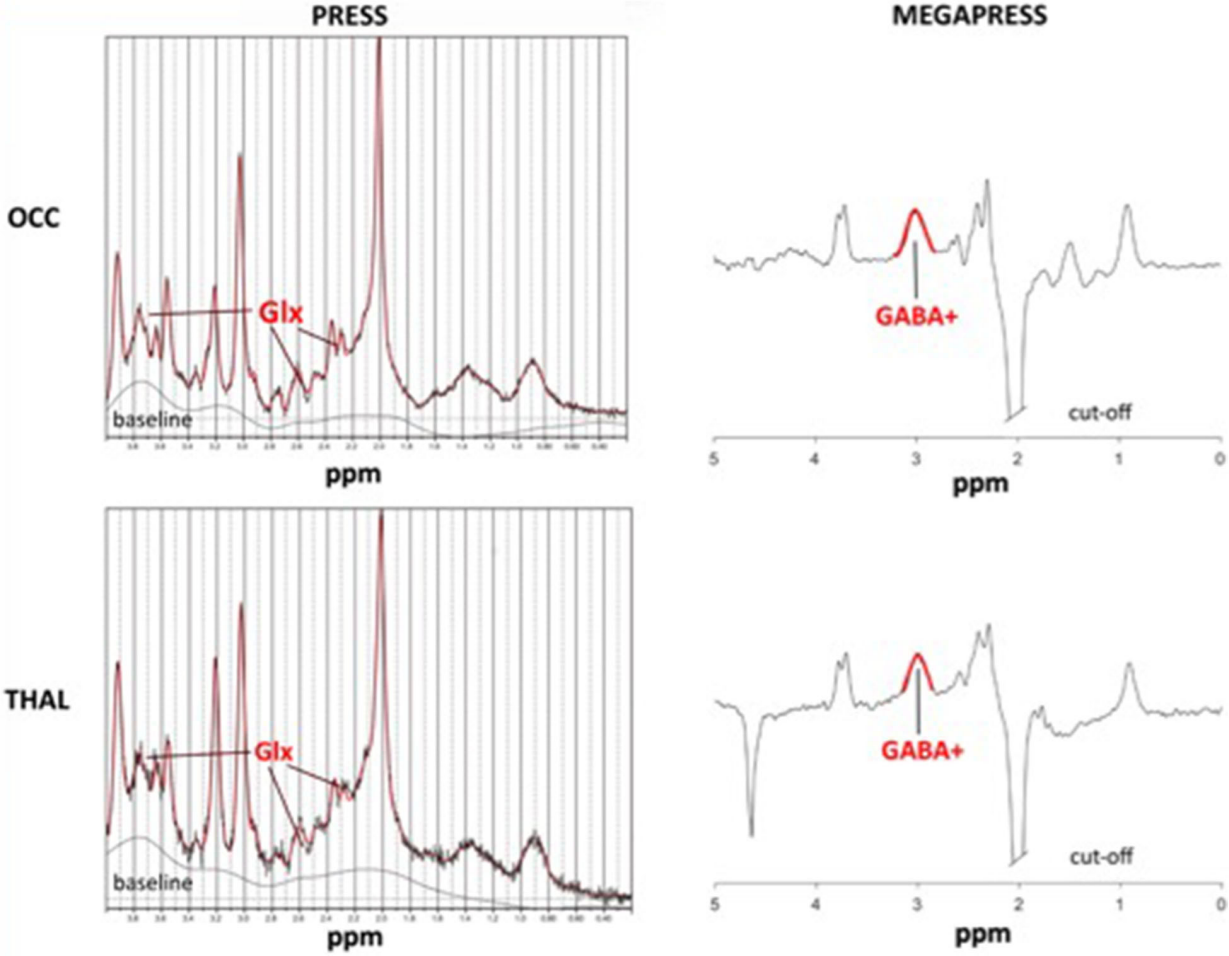
Example spectra of PRESS & MEGAPRESS measurement in the occipital cortex and right thalamus. Abbreviations: Example spectra of a single patient using PRESS (left) and MEGAPRESS (right), with voxel placement in the occipital cortex (upper row) and right thalamus (bottom row). GABA = gamma-amino-butyric acid, GLX = glutamate/glutamine, MegaPress = Mescher-Garwood-Point Resolved Spectroscopy, OCC = occipital cortex, PRESS = Point-RESolved Spectroscopy, ppm = part per million, THAL = Thalamus

^1^H-MRS was performed using a Philips 3.0 T Achieva X-series scanner with a 32-channel head coil. The MRI session consisted of a high resolution T1-weighted scan (MPRAGE, TR/TE: 8.5/3.9 ms, voxel size (1 mm)^3^ isotropic, FOV 240 × 240 × 220 mm) allowing an accurate voxel placement, as well as PRESS and MEGA-PRESS scans of the occipital cortex (3 × 3 × 3 cm^3^, centered around the midline projecting to the primary visual cortex), and the right thalamus (3 × 3 × 2.5 cm^3^). For the thalamic voxel the medial edge was aligned with the third ventricle, its anterior edge with the most anterior point of the thalamus. Of note, the thalamic voxel included to some degree the right globus pallidus and putamen in the average subject (for voxel placement and representative spectra see also Figs. 1 and 2).

PRESS sequences used here had the following parameters: TR/TE: 2000/30 ms, spectral bandwidth of 2 kHz, sampling rate of 2048 points, 90-degree flip angle, number of signals averaged = 32. The MEGA-PRESS sequence used a longer TE (TR/TE: 2000/68 ms) and contained a 14-ms sinc-gaussian ON editing pulse at 1.9 ppm and an OFF editing pulse at 7.46 ppm (spectral bandwidth of 2 kHz and a sampling rate of 2048 points). A total of 320 transients were acquired: 10 averages of 16 ON and 10 averages 16 OFF scans. For both Point-RESolved Spectroscopy (PRESS) and MEGA-PRESS sequences, a PB-auto second order shim was used to reduce field inhomogeneities and water suppression was accomplished using VAPOR (80 Hz window). Macromolecules were not suppressed; therefore, GABA in this study refers to GABA+ macromolecules (GABA+).

Glutamate/glutamine (GLX), N-acetylaspartate (NAA), and creatine (Cr) concentrations from the PRESS acquisition were quantified using LCModel’s (version 6.3) standard basis set including water scaling. Of note, since there is no Cr in the CSF, the metabolite to Cr ratio serves the additional purpose of tissue correction, which is an alternative to referencing the metabolite in question to water with an additional correction for tissue volume (gray and white matter). As Cr and phosphocreatine (PCr) cannot be resolved reliably at 3 T, Cr_total_ here refers to Cr and PCr.

After metabolite quantification, we assessed spectral quality before performing statistical analyses, e.g. group comparison. First of all, spectra were inspected visually, in some cases no metabolite signal could be detected and data were excluded from further analyses. Additionally, LCModel allows for the estimation of signal to noise ratios (SNRs). SNR is defined as the ratio of the maximum in the spectrum minus the baseline (within the analysis window) to twice the root mean square of the residuals. If spectra displayed a SNR > 20, resulting metabolites with a %SD < 15 were submitted to further statistical analyses.

GABA+ and Cr_total_ concentrations were acquired using MEGA-PRESS and quantified from the difference (ON – OFF) and OFF spectra, respectively, using GANNET version 2.0, a GABA Analysis Toolkit (http://www.gabamrs.com). After correcting for any frequency drifts using changes in peak Cr_total_ frequency as a reference, GANNET fits a Gaussian + baseline fit to the averaged ON-OFF difference spectra and uses the area under the curve to determine the GABA+ concentration (i.u.). Again, Cr_total_ served as an internal reference to control for interindividual differences in tissue volume; Cr_total_ was quantified using a Lorentz-Gaussian lineshape to fit the averaged OFF spectrum, where again the area under the curve quantifies the Cr concentration in the voxel, yielding GABA+/Cr_total_ ratios.

### Statistical analysis

Mann-Whitney tests (MWU) were performed on each MRS measure [GABA+/Cr, GLX/Cr_total_, Cr/H2O, (NAA + NAAG)/Cr] separately for each of the two MRS voxel regions (THAL and OCC) to detect differences between migraneurs and HCs. Relationships between local GABA and/or GLX concentrations and pain measures were determined using a Spearman correlation. Statistical significance was set at *p* < .05.

## Results

Originally 19 migraineurs and 18 healthy controls were scanned using PRESS and MEGA-PRESS sequences. After careful examination 4 migraine patients were removed for the following reasons: One patient due to the presence of ninety headache days during the observation period making a chronic migraine or medication overuse headache the likely diagnosis, two patients due to a lack of any migraine attacks during the observation period, and one due to recurrent aura symptoms. Exclusion of four migraine patients did not affect the group statistic: Fifteen migraine patients (12 females, 3 males; age 35.2 ± 10.8 years) and fifteen control subjects (12 females, 3 males, age 33.4 ± 8.5 years, *p* > 0.7)) were included in the statistical analyses. Quality control of the MRS spectra excluded 4 patients and 3 HCs from the GABA thalamus, GLX thalamus and GLX occipital analyses, as well as 2 patients and 1 HC from the GABA occipital voxel.

Within the migraine group both average pain intensity and number of headache days were both positively correlated with the MIDAS score (*r* = 0,54, *p* = 0,037; and *r* = 58, *p* = 0.024 respectively). There were no significant correlations between pain measures and BDI scores (*p* > .05).

MWU tests revealed higher levels of Glx/Cr_total_ in both the THAL (z = 2.54, *p* = .011) and OCC (z = 2.08, *p* = .038) voxel locations (Table 2, Fig. 1). Levels of GABA/Cr_total_, Cr/H20, and NAA/Cr_total_ were similar between patients and HCs. Correlation analyses within the migraine group revealed no significant correlations between GLX/Cr_total_ and/or GABA+/Cr_total_ in either of the two brain regions with pain intensity or MIDAS scores (*p* > .05).

**Table 2:** Metabolite concentrations

MWU - Test: Patients vs. Controls									
		Rank Sum					Z	Valid N	Valid N
Variable	Region	MIGA	Control	U	Z	*p*-value	adjusted	Patients	Control
GABA+/Cr	THAL	136	140	62	0,215	0,829	0,215	11	12
	OCC	180	198	89	−0,073	0,942	−0,073	13	14
Glx/Cr	THAL	177	99	21	2739	0,006	2739	11	12
	OCC	166	110	32	2062	0,039	2062	11	12
Cr/H2O	THAL	134	119	53	0,460	0,646	0,460	11	11
	OCC	167	184	76	-0,410	0,682	-0,410	13	13
(NAA + NAAG)/Cr	THAL	131	122	44	1022	0,307	1022	10	12
	OCC	133	143	65	0,031	0,975	0,031	11	12

*GLX* glutamate/glutamine, *GABA* gamma-amino-butyric acid, *NAA* N-actetyl-aspartate, *Cr* creatine

## Discussion

The purpose of this study was to further elucidate the role of the neurotransmitters GLX and GABA in migraine. We specifically investigated local GLX and GABA concentrations in the occipital lobe including the primary visual cortex, and the right thalamus of migraine patients and HCs. Migraine patients had increased GLX levels in both the occipital cortex and the right thalamus, but showed no group differences in GABA in these two regions. Within the migraine group, no association between neurotransmitter concentrations and pain intensity or disability scores were found.

Our results support recent findings indicating that higher regional GLX levels are linked to the presence of migraine [13]. Zielmann et al. could demonstrate in a larger cohort (at a field strength of 7 T) that specifically glutamate levels were increased in the occipital cortex of patients with migraine [12]. Interestingly the effect was only observed in patients with migraine without aura, but not in patients with migraine with aura. Increased glutamatergic activity has been hypothesized to play an important role in the pathophysiology in migraine for a long time, specifically that increased glutamate levels lead to a cortical hyperexcitability within sensory cortices [21]. Of note, other studies applying spectroscopy in migraine could not confirm increased GLX levels, for example when looking at cerebellar GLX in patients with familiar hemiplegic migraine [22, 23]. Against this background one might argue that altered GLX levels are either region dependent or, from a statistical point of view, that the cohorts investigated so far are too small to consistently identify subtle differences.

The thalamus, our second region of interest, is known to play a crucial role in relaying not only nociception, but almost all sensory modalities [17, 18]. During migraine attacks most patients, apart from pain, additionally suffer from photo- and phonophobia. Given that hyperexcitability also plays a role in the genesis of multi-sensory symptoms during a migraine attack, this might either be a common phenomenon across different sensory cortices or one might postulate hyperexcitability within a structure with sensory input to the sensory cortices, i.e. the thalamus. Although at the current stage one can only speculate on the origin and spread of the pathophysiological processes leading to the clinical outbreak of a migraine attack, our data at least provide evidence for thalamic involvement in terms of an interictal increase of glutamatergic activity.

Interestingly, increased GLX levels have also been observed in other pain conditions [24]. Harris et al., for example, found elevated GLX levels in the insular cortex of fibromyalgia patients [25], while Fayed et al. described increased GLX level in the posterior cingulate cortex of patients with fibromyalgia and somatization disorder [26], suggesting that GLX plays a role in the pathogenesis of chronic pain. To date, only a few studies have looked at thalamic GLX in chronic pain conditions and the results are not yet conclusive [27, 28]. Our group has recently shown a link between GLX and pain sensitivity in healthy subjects where subjects more sensitive to pain stimuli had higher level of GLX in a network of brain regions known to play a role in pain perception, including the thalamus [29]. Against this background it is tempting to hypothesize, that there is a link between pain sensitivity and also the development of either chronic or recurrent pain conditions on the one hand and GLX levels in brain regions related to sensory and nociceptive processing on the other hand. However, this needs to be further elucidated; for now our results indicate increased interictal GLX levels in both the occipital cortex and the right thalamus in patients with migraine, supporting the notion of an extended network displaying cortical hyperexcitability.

With respect to GABA, several studies have investigated local GABA concentrations in migraine patients. Bigal et al. reported no significant differences in occipital GABA concentrations between migraine patients and HCs [16]. When pooling the migraine groups, patients with a higher disease burden had lower occipital GABA levels than patients with a lower disease burden. Using the MEGA-PRESS technique, Bridge et al. reported decreased GABA levels in the occipital cortex in migraine patients with aura [15], whereas Aguila et al. reported higher GABA levels in migraine patients in the posterior cingulate [14]. A later investigation of the same cohort could show that pain measures were negatively correlated with GABA concentrations. Our data are in line with the study of Bigal et al., indicating that migraine patients without aura display no altered GABA levels in the occipital cortex [16].

Taking all studies investigating local GABA concentrations in migraine patients into account, the results are not yet conclusive. The studies conducted so far looked at similar group sizes and age ranges, as well as cohorts with female predominance. Furthermore, all patients were investigated interictally. However, there were differences with respect to data acquisition, especially voxel placement, and migraine subtypes. As outlined above, our data do not support the notion of altered GABA levels in the occipital lobe of patients with migraine without aura and we speculate that altered GABA levels in that region might either be specific to migraine with aura or a characteristic of the migraine attack, but not of the interictal state.

Importantly, GABA has been found to be decreased in several other pain conditions such as fibromyalgia, diabetic neuropathic pain and trigeminal pain. Decreased GABA levels were found in the insular cortex of fibromyalgia patients [30] and patients with diabetic pain [24], while decreased thalamic GABA levels were found in patients with neuropathic pain after spinal trauma [31] and patients with neuropathic trigeminal pain [32]. As such it will be of interest to also look at GABA levels in other brain region related to nociceptive processing and multisensory integration, such as the insular cortex and cingulate cortex, in migraine patients.

## Limitations

There are several limitations in our study that need to be pointed out. One drawback of this and other studies is the cross-sectional nature at a time point outside the actual migraine attack. As we measured GLX interictally, it cannot be derived from our data, if the elevated GLX level is a rebound phenomenon of a migraine attack, or if it is permanently elevated and facilitates nociceptive transmission. It will be of major importance to assess GLX and GABA levels during a migraine attack (or the day before), with the option to also account for laterality effects, or even longitudinally in- and outside a migraine attack to assess dynamic changes in neurotransmitters as they related to the pain event. Furthermore, with respect to headache frequency the group was rather heterogeneous, with number of headache days ranging from 2 to 40 (within 3 months). In future studies it will be important to investigate more homogeneous (and larger cohorts) to capture potential associations between neurotransmitter ratios and disease burden.

Another drawback from a methodological point of view is that GLX reflects both glutamate and glutamine levels. One might criticize that our interpretation of local hyperexcitability is based on the assumption that increased GLX levels are primarily caused by glutamate rather than glutamine, which is not necessarily the case. However, glutamate is one of the major components of the glutamic acid cycles taking place in both astrocytes and neurons, as such GLX is likely to also reflect the glutamate pool and/or turn around. Higher field strengths hold promise to separate both metabolites which will be helpful to further shed light onto the underlying pathophysiology [12].

## Conclusion

We report increased GLX levels in the right thalamus and occipital cortex in migraineurs, but no changes in local GABA levels, supporting an extended network of cortical hyperexcitability.

We propose that thalamic GLX plays a role in the pathophysiology of migraine, but the full relevance of both neurotransmitters remains to be elucidated. Further research is needed to investigate the role of GLX-GABA-ratios in more depth, particularly over time and within larger cohorts, with migraineurs with and without aura and possibly other subgroups, since different pathomechanisms might play a role in migraine subtypes. It will also be interesting to relate thalamic metabolite levels during the migraine attack not only to headache intensity, but also to decreased pain thresholds, as well as phono- and photophobia, symptoms often present during migraine attacks, indicating altered multisensory perception.

## Abbreviations

BDI: Beck depression inventory
Cr: Creatine
CSF: Cerebrospinal fluid
CSF: Cerebrospinal fluid
GABA: gamma-amino-butyric acid
GLX: Glutamate/glutamine
HC: Healthy control
H-MRS: Proton magnetic resonance spectroscopy
H-MRS: Proton magnetic resonance spectroscopy
MegaPress: Mescher-Garwood-Point Resolved Spectroscopy
MIDAS: Migraine Disability Assessment
MWU: Mann-Whitney-U
NAA: N-actetyl-aspartate
OCC: Occipital
PCr: Phosphocreatine
PRESS: Point-RESolved Spectroscopy
THAL: Thalamus
